# Supplementary figures and images for: Differential responses to folic acid in an established keloid fibroblast cell line are mediated by JAK1/2 and STAT3
Source: PLoS One. 2021 Mar 4;16(3):e0248011. doi: 10.1371/journal.pone.0248011 (PMC7932104; doi:10.1371/journal.pone.0248011)

Supplemental Figure 1

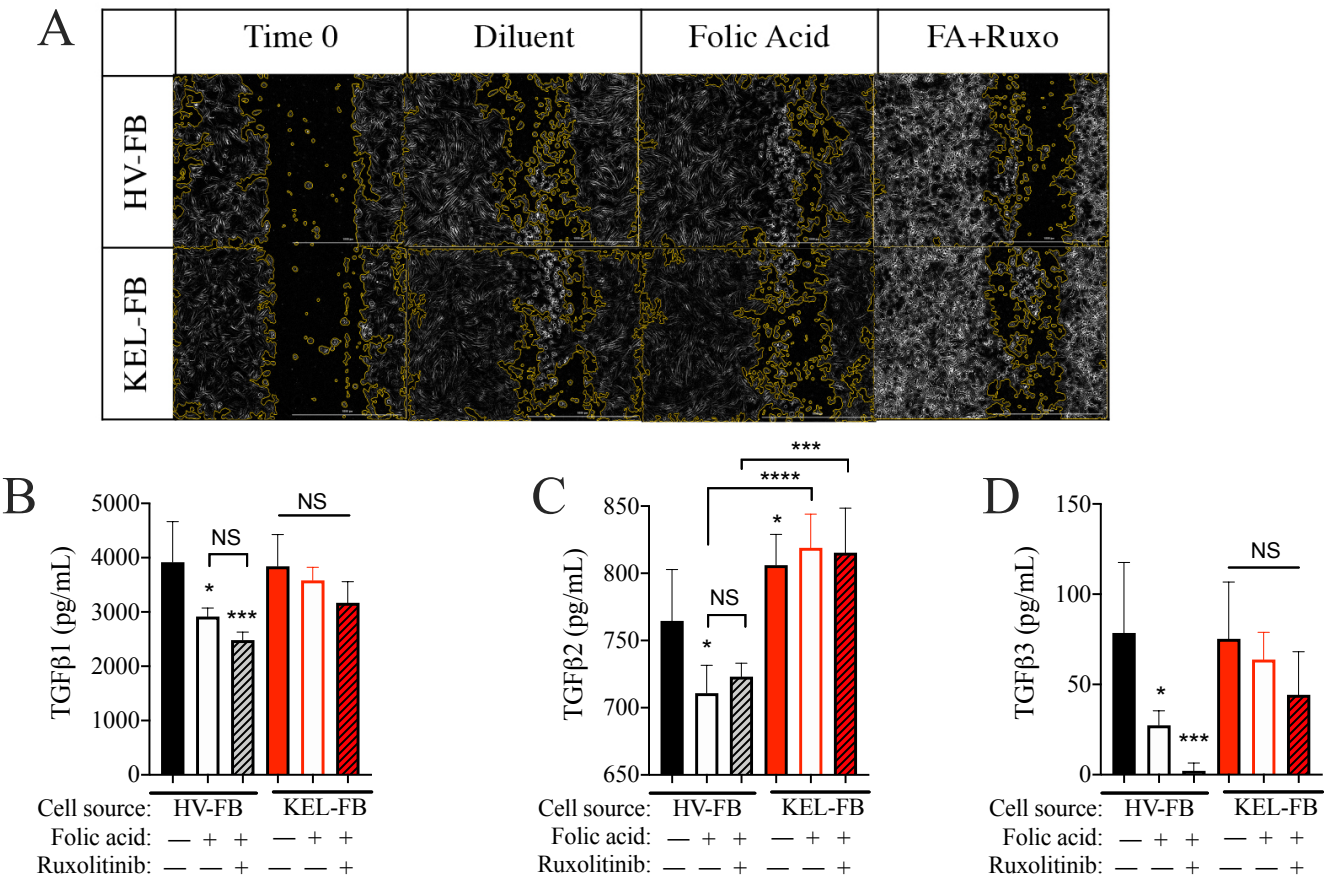

Supplement: S1 Fig — (A) Representative scratch assay image after 12 hours for fibroblasts (FB) cell lines from a healthy volunteer (HV-) or keloid scar (KEL-) treated with folic acid (FA) and ruxolitinib (Ruxo). Masking performed by Scratch App (BioTek). (B and C) Supernatant levels for TGFβ1, 2, and 3 for FB treated with folic acid and ruxolitinib. Results are representative of two independent experiments and displayed as mean ± SEM for triplicate wells per condition. NS = not significant; * = p <0.05; *** = p < 0.001, **** = p < 0.0001 versus HV with diluent condition unless otherwise indicated as determined by ANOVA with Sidak adjustment. (PDF) [file pone.0248011.s001.pdf]
